# Uptake and Retention of Nanoplastics in Quagga Mussels

**DOI:** 10.1002/gch2.201800104

**Published:** 2019-07-02

**Authors:** Rachel L. Merzel, Lauren Purser, Taylor L. Soucy, Monica Olszewski, Isabel Colón‐Bernal, Melissa Duhaime, Ashley K. Elgin, Mark M. Banaszak Holl

**Affiliations:** ^1^ Chemistry Department University of Michigan Ann Arbor MI 48109 USA; ^2^ Ecology and Evolutionary Biology University of Michigan Ann Arbor MI 48109 USA; ^3^ NOAA Great Lakes Environmental Research Laboratory Lake Michigan Field Station Muskegon MI 49441 USA; ^4^ Chemical Engineering Monash University Clayton VIC 3800 Australia

**Keywords:** AFM‐IR, microplastics, mussels, nanoplastics, PT‐IR

## Abstract

Here, a set of experiments to assess the feasibility of using an invasive and widespread freshwater mussel (*Dreissena rostrformis bugensis*) as a sentinel species for nanoplastic detection is reported. Under laboratory experimental conditions, mussels ingest and retain fluorescent polystyrene (PS) beads with carboxylic acid (—COOH) termination over a size range of 200–2000 nm. The number of beads the mussels ingested is quantified using fluorescence spectroscopy and the location of the beads in the mussels is imaged using fluorescence microscopy. PS beads of similar size (1000–2000 nm) to mussels' preferred food are trafficked in the ciliated food grooves of the gills. Beads of all sizes are observed in the mussels' digestive tracts, indicating that the mussels do not efficiently reject the beads as unwanted foreign material, regardless of size. Fluorescence microscopy shows all sizes of beads are concentrated in the siphons and are retained there for longer than one month postexposure. Combined atomic force microscopy–infrared spectroscopy and photothermal infrared spectroscopy are used to locate, image, and chemically identify the beads in the mussel siphons. In sum, these experiments demonstrate the potential for using mussels, specifically their siphons, to monitor environmental accumulation of aquatic nanoplastics.

## Graphical abstract

**Can quagga mussels (Dreissena rostriformis bugensis), a widespread and invasive freshwater species** that alters local ecosystems, act as a sentinel species for detecting nanoplastics? In the laboratory, mussels ingest and retain 200–2000 nm fluorescent polystyrene beads, which are in the size range for the mussels' preferred food and are trafficked like food in the ciliated grooves of the gills.

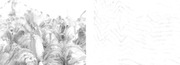

## 1 Introduction

Aquatic microplastics are currently the focus of intense research efforts and are generally recognized as a substantial problem due to their pervasiveness, persistence in the environment, and potential toxicity.^1^, ^2^, ^3^, ^4^, ^5^, ^6^, ^7^, ^8^, ^9^ Microplastics are of such interest and concern because they are ingested by aquatic organisms, either unintentionally or when they are mistaken for food, for example algae or plankton of similar size.^10^, ^11^, ^12^ When ingested, they have the potential to disrupt physiological processes in aquatic life and biomagnify up the food chain, including into humans.^13^, ^14^, ^15^, ^16^, ^17^ As such, there have been substantial efforts to characterize the concentrations, identities, and sources of aquatic microplastics. The term “microplastic” technically refers to plastics over the micrometer size range: 1–1000 µm. However, the term has been ascribed operationally to a variety of size ranges, including 333 µm to >5000 µm,^18^ 106 µm to >4750 µm,^8^ anything smaller than 1 cm (10 000 µm)^3^ and anything smaller than 5 cm.^9^ The focus on larger microplastics is not surprising given the challenges associated with analyzing smaller plastics, as highlighted in this article.

Mussels have been used as a sentinel species for monitoring pollution since they are filter feeders common in both freshwater and marine environments, are an important part of the food web, and are consumed by humans.^16^, ^19^, ^20^, ^21^ The blue mussel (*Mytilus edulis*) has been used to monitor the presence of metal, halogenated hydrocarbon, organotin, and pharmaceutical species in the marine environment for over two decades and more recently has been used to explore microplastic exposure, as recently reviewed extensively by Beyer et al. and in references therein.^19^ Quantitative laboratory studies as well as environmental studies of polystyrene (PS) and polyethylene sphere uptake in mussels (*M. edulis*) have demonstrated that these materials are readily taken up by mussels and that the plastics can be transferred to other creatures that eat them, including crabs (*Carcinus maenas*) and lugworms (*Arenicola marina*).^12^, ^22^, ^23^ Fewer studies have been conducted using freshwater mussels (but see Magni et al.^24^—though this experiment spanned less than a week). Although the problem of microplastic exposure and uptake is well‐documented, there is still much work to be done to understand the impacts on the environment and human health, particularly in freshwater systems and over extended observational periods.^25^

Even with this focus on microplastics, there has been very little research on aquatic nanoplastics, which can either enter the environment by direct release or by degradation of larger plastics.^26^, ^27^ The lack of attention has primarily been due to the fact that nanoplastics are difficult to isolate and characterize; because they are so small, the standard isolation and characterization techniques used to study microplastics cannot be used for nanoplastics. Using conventional filtration to isolate aquatic nanoplastics would be time and cost prohibitive. Thousands to tens of thousands of liters of water would have to be filtered through nanoscale sized pores in order to acquire statistically relevant quantities of environmental aquatic nanoplastics. We hypothesize that techniques such as centrifugation with density gradients are likely more efficient. If isolated nanoplastics are present at a sufficient concentration, it may be possible to characterize them with conventional spectroscopy techniques (e.g., Raman or Fourier transform infrared spectroscopy). However, based on our calculations presented in this study, aquatic nanoplastics are usually not present in high enough concentrations for these analytical techniques to be useful. Therefore, scanning probe or electron microscopy must be used, which creates a “needle in a haystack” problem when hunting for the nanoplastics on a surface at the millimeter or greater scale.

For these reasons, the environmental concentration of nanoplastics has been difficult to quantify, and even the environmental concentration of microplastics is low (≈1–10 particles per 100–1000 L) for the analytical techniques necessary to use for nanoplastics.^8^, ^9^ We took advantage of the natural concentrating ability of filter feeding mussels to study nanoplastic accumulation. We used quagga mussels (*Dreissena rostriformis bugensis*), a freshwater invasive species with a broad distribution across North America and Europe that greatly alters local ecosystems, primarily due to their ability to efficiently filter phytoplankton out of the water.^28^, ^29^ As widespread invasive species, dreissenid mussels (which include quagga mussels and their close relative, zebra mussels (*D. polymorpha*)) are therefore available for collection and analysis of nanoplastics in many regions of the world. Dreissenid mussels filter between 1 and 7 L (depending on species, mussel size, season, and water temperature) of water per day,^30^ and it is well known that mussels can selectively accept or reject microscale objects they take in.^31^, ^32^, ^33^, ^34^ Browne et al. reported that once 2–16 µm PS microplastics are ingested, they can translocate from the digestive system into the circulatory system and remain in blue mussels for up to 48 d.^35^ Interestingly, smaller microplastics (3.0 µm) moved into the circulatory system more quickly than larger nanoplastics (9.6 µm). In zebra mussels (*Dreissena polymorpha*), PS microbeads were concentrated in the tissues, gut lumen, and hemolymph after 6 d of exposure.^24^ These studies have focused on the uptake, selection, sorting, and physiological effects of microplastics on mussels, but mussel uptake of nanoplastics, and more generally the effect of nanoplastics on aquatic ecosystems, has largely not been investigated.^26^

To that end, we carried out experiments using quagga mussels collected from offshore regions of the Laurentian Great Lakes, USA. Our goal was to assess the extent to which mussels ingest and retain nanoscale PS beads—an appropriate model material, as PS (typically Styrofoam) is commonly found in aquatic environments. We designed a series of proof‐of‐concept studies to determine where to look in the mussel body for retained environmental nanoplastics in mussels collected in situ. We exposed the mussels to nanomolar concentrations of carboxylic acid‐terminated fluorescent PS beads. The concentrations of nanoplastics used in this study are likely higher than those expected in open water, but are likely comparable to areas near the outflow of wastewater treatment facilities.^36^ We sought to determine whether dreissenid mussels could serve as a sentinel species for monitoring aquatic nanoplastics,^19^ given the challenges mentioned above of isolating and characterizing nanoplastics. Beads with carboxylic acid termination were used because chemical weathering from UV radiation results in surface oxidation of the plastic.^37^ Importantly, oxidation resulting from UV radiation increases plastic degradation, supporting the hypothesis that aquatic nanoplastics can originate from microplastics. Following exposure to the PS beads, the mussels were dissected and their organs fluorescently imaged. Clearance of nanoplastics was monitored using fluorescence microscopy until the feces and pseudofeces were no longer fluorescent (21–44 d). The retained nanoplastics in the mussel organs were quantified using fluorescence spectroscopy. The experiments presented here provide quantitative measures of the relative rates of uptake and excretion of PS beads by quagga mussels.

These results are particularly interesting when viewed in context of previous work by Morton^34^ on mussel anatomy and feeding mechanisms, which has been followed up on with extensive food trafficking studies.^31^, ^32^, ^33^, ^38^, ^39^, ^40^, ^41^, ^42^, ^43^ In general, these studies show how mussels move ingested particles on the ctenidium, or gills. There is strong evidence from a number of these studies demonstrating the ability of mussels to qualitatively differentiate and selectively expel particles at a number of points along the digestive pathway. However, the results presented here demonstrate that with 200–2000 nm PS beads, the mussels did not effectively discriminate nanoplastic from food and moved the beads entirely through the digestive tract. Furthermore, the mussels retained in the range of 10^7^–10^8^ beads, which in addition to impacts on mussel health, raises concerns of bioaccumulation.

## 2 Results and Discussion

### 2.1 Mussels Ingest Polystyrene Beads

**Figure 1:**
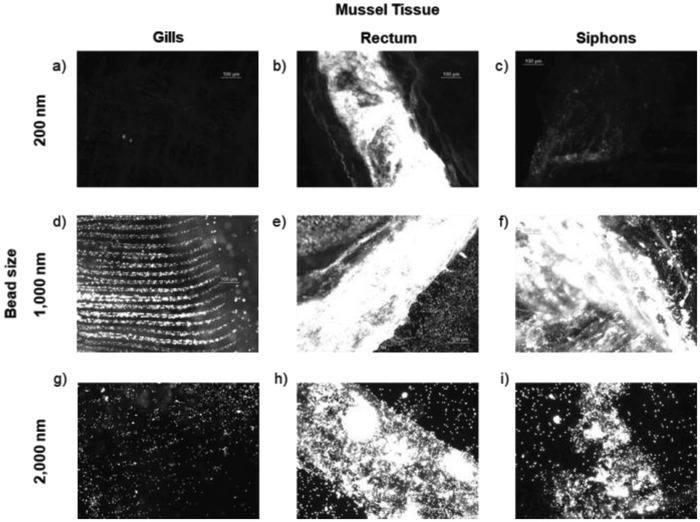
Exemplar fluorescence images of isolated tissues (gills, rectums, siphons) from quagga mussels dosed with 200 nm (a–c), 1000 nm (d–f), and 2000 nm (g–i) carboxylate‐modified PS beads containing a red dye (excitation/emission 580/605). All images were acquired with the same microscope settings, leading to some images appearing overexposed. The images demonstrate the substantial accumulation of beads of all three sizes in the rectums and of the 1000 and 2000 nm beads in the siphons. Mussels were dosed with 200 and 1000 nm beads at 1 × 10^−12^ m and 2000 nm beads at 0.01 × 10^−12^ m for 24 h.

Particularly with the 1000 nm PS beads, fluorescence microscopy demonstrated that the mussels move the beads through the gills in the same manner as described for food particles (**Figure** **1**).^34^ Figure 1d shows patterning of the beads in the ciliated grooves of the gills. The larger 2000 nm beads also showed some of this same patterning, in addition to bead aggregation (Figure 1g), but this effect was not observed with the 200 nm beads. These differences in trafficking through the gills as a function of bead diameter are not unexpected: the *Nannochloropsis* fed to the mussels is 1500–2000 nm in diameter and given at a concentration of about 0.1 to 0.001 × 10^−12^ m. With the exception of the images with 2000 nm beads, the images shown in Figure 1 are from dosing experiments carried out at 1 × 10^−12^ m. We also completed 24 h dosing studies with 200 and 1000 nm beads at 0.1 and 0.01 × 10^−12^ m (Figure S1, Supporting Information, for exemplar images). At these lower concentrations, little to no fluorescence was evident in most of the organs, especially with the 200 nm beads. At 0.01 × 10^−12^ m with the 1000 nm beads, substantial fluorescence was evident in the siphons, a phenomenon explained in more detail below.

Fluorescence spectroscopy was used to quantify the number of PS beads the mussels took up (Table S1, Supporting Information). Overall, the mussels ingested and retained one to two orders of magnitude fewer beads after 24 h as compared to the PS bead concentration in the culture water. In 24 h, three mussels took up (mean ± S.D.) 6 × 10^8^ ± 3 × 10^8^ 1000 nm PS beads dosed at 1 × 10^−12^ m (6 × 10^10^ PS beads in the culture water). With 200 nm beads at 1 × 10^−12^ m, two mussels took up an average of 5 × 10^9^ beads. When exposed to 2000 nm beads at 0.01 × 10^−12^ m (6 × 10^8^ beads in the water), three mussels each took up an average of 1 × 10^7^ ± 6 × 10^5^ beads. Individual variation in filtering rate could explain the difference in the number of beads taken up. It should be noted that the mussels in each treatment group were housed in the same beaker and were, therefore, in competition for the same beads. It is possible that intense filtering by one mussel could have influenced the uptake by other mussels. However, assuming relatively similar uptake and filtration rates by all three mussels in the beaker, the mussels would have cleared the water if they retained all the beads they took up.

Another point we considered is that the beads may settle and sediment over time. However, it is likely to only have a minimal effect, if any at all, on the experiment and results. The density of the PS beads is 1.05 g cm^−3^, making them only slightly negatively buoyant. Due to the dye, the PS beads are brightly colored, and we can observe when they settle, for example, in the sample bottle. We did not observe settling over the time courses of the experiments (24–72 h). Furthermore, mussels create their own microcurrents when filtering and may have had the ability to stir up any settling beads.

These data raise the question of whether mussel uptake of nanoplastics is a concern at environmental levels of nanoplastics pollution. A 2016 study by Sutton et al. reported an average of 700 000 microplastic particles km^−2^ in surface water.^9^ In a 2017 paper, Cable et al. reported concentrations of particles ranging from ≈126 000 to 2 000 000 particles km^−2^.^8^ We converted this estimate to a volume‐based concentration, which is on the order of magnitude of one particle in 100 L of water (Figure S2, Supporting Information). Assuming mussels filter 6 L d^−1^, it would take 4.5 million years for a mussel in the wild to reach a concentration of 10^8^ beads. Therefore, if nanoplastics concentration is on the same order as reported microplastic concentration, uptake is unlikely to cause problems or be a pressing concern. If local nanoplastic concentrations are higher, either due to release from waste water treatment plants, higher concentrations in the benthos where mussels reside, or as a result of microplastic degradation or fragmentation,^3^, ^27^, ^36^ then it is conceivable that ingestion of nanoplastics could pose a problem. For example, Figure S3 (Supporting Information) demonstrates that one 50 µm (50 000 nm) particle could fragment into 1.25 × 10^5^ 1000 nm particles or 1.0 × 10^9^ 50 nm particles. This analysis still assumes one microplastic in 100 L, but locally higher microplastic concentrations could result in very high concentrations of nanoplastics. If the nanoplastic concentration is on the same order as the microplastic material load, bioaccumulation of nanoplastics could occur depending on relative uptake versus excretion rates (as discussed in more detail below).

Mussels use particle sorting mechanisms at several stages along the digestive tract, and dreissenid mussels can filter particles larger than 700 nm out of the water.^32^ Ingested material not immediately removed from the mantle cavity through the inhalant siphon as pseudofeces is transported through the gills to the mouth.^33^, ^34^ Microplastics that are ingested there tend to accumulate in the gut. However, the mussels have secondary sorting mechanisms in the stomach, and rejected material is moved through the mid‐gut and excreted via the anus and exhalant siphon. Material accepted into the digestive gland is phagocytosed, but if it proves to be indigestible, it is excreted via the pericardial gland and excretory organs. It is possible that the phagocytosis could be due to the negative surface charge of the carboxylic acid‐terminated PS beads. However, using the negatively charged PS beads is a good model for environmental nanoplastics which undergo oxidation due to chemical weathering from UV radiation.^37^ The nanoplastics that mussels in the environment encounter are likely negatively charged, as well.

These processes of isolating and excreting unwanted particles are not perfect. It has been reported that once particles are ingested into the gut of the blue mussel, they can translocate into the hemolymph and remain in the circulatory system for over a month and a half.^35^ This finding is consistent with our results on mussel retention and clearance of PS beads.

With this previous work as context, the physiological pathways by which the PS beads are moving through the mussels are unclear. The presence of the beads in the rectum could be due to (1) rejection in the stomach; (2) rejection in the digestive gland after phagocytosis and being determined indigestible; or (3) transport all the way through the digestive tract as food. Regardless, our results demonstrate that mussels are not immediately able to reject PS beads in their particle sorting processes. The retention of 200 nm particles in the rectums of the mussels (Figure 1b) is not necessarily inconsistent with studies by Sprung and Rose in which mussels only retained particles larger than 700 nm.^32^ The samples in their studies were passed through a 450 nm membrane filter that would have removed particles smaller than this pore size; therefore, no 200 nm particles were present. Additionally, these experiments largely examined the gills of the mussels and what remained in the water but did not directly image the digestive and excretory organs. Our experiments show that the mussels do in fact take up smaller particles, but we only saw substantial concentration in the rectum. As we did not isolate hemolymph for analysis, we cannot assess the extent to which some of the PS beads are translocating out of the digestive tract and into the hemolymph, as was observed in the work of Magni (quagga mussels)^24^ and Browne (blue mussels).^35^ However, following dissection of the quagga mussels in our study, the fluid from the mantle did brightly fluoresce and individual beads were visible.

Regardless of the fate of the beads within the mussel, our fluorescence images show that the mussels do not immediately reject the beads upon bringing them in through the inhalant siphon. In fact, as discussed in the next sections, the mussels cannot clear all of them over 45 d. This leads to concerns of bioaccumulation if the mussels cannot eventually clear the PS material.

### 2.2 Mussels Cleared the Majority of the PS Beads

**Figure 2:**
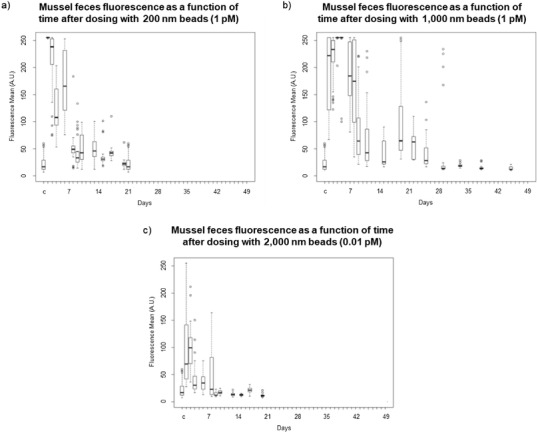
Box plots of the mean fluorescence intensity of mussel feces following dosing with PS beads containing a red dye. Mussels were dosed with a) 200 nm beads at 1 × 10^−12^ m and allowed to clear for 21 d; b) 1000 nm beads at 1 × 10^−12^ m and allowed to clear for 44 d; and c) 2000 nm beads at 0.01 × 10^−12^ m and allowed to clear for 20 d. The boxes labeled “c” are the control.

Fluorescence microscopy was used to monitor the clearance of the beads; mussel feces from all three mussels kept in the beaker were collected together and combined at regular intervals and imaged. Table S2 (Supporting Information) summarizes the clearance studies and Figure S4 (Supporting Information) shows exemplar images of the feces. **Figure** **2** shows boxplots illustrating the trends in fluorescence intensity over time.

**Figure 3:**
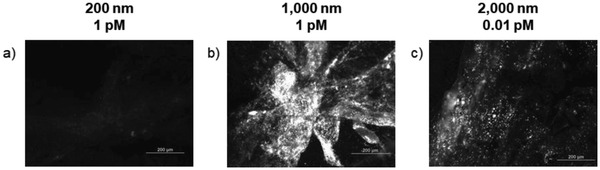
Fluorescence images of quagga mussel siphons after mussels were dosed with a) 200 nm (1 × 10–12 m); b) 1000 nm (1 × 10–12 m); and c) 2000 nm (0.01 × 10–12 m) carboxylic‐acid‐terminated PS beads with a red dye and allowed to clear until their feces were no longer fluorescent (see Figure 2 for clearance times).

With the 1000 and 2000 nm beads, residual fluorescence was observed in the organs after the feces were no longer fluorescent. For example, **Figure** **3** shows fluorescence in siphons after the beads were no longer being eliminated through these mussels' feces and pseudofeces. In the experiments with the 1000 nm beads, the feces were no longer fluorescent and the mussels were dissected at 44 d (Figure 2b and Figure S4, Supporting Information). This is in line with the work of Browne et al.^35^ and Magni et al.,^24^ who demonstrated that PS particles in mussels translocate from the gut to the circulatory system and persist in the mussel for 48 d.^35^ The residual fluorescence observed in the mussels can likely be attributed to PS beads that became lodged in the mussel tissues and so could not be cleared via the circulatory system (Figure 3b and Figure S5c,d, Supporting Information). The beads remaining in the gills were not in the ciliated food grooves but were distributed through the rest of the mussel body. The digestive tract displayed markedly lower fluorescence after being allowed to clear. The substantial fluorescence signal and accumulation of the PS beads in the siphons are discussed further in the below. The mussels dosed with 2000 nm beads at 0.01 × 10^−12^ m were dissected after clearing for 20 d (Figure 2c). In the 2000 nm study, there were already two orders of magnitude fewer beads to clear as compared to the experiment with 1000 nm beads (0.01 × 10^−12^ m vs 1 × 10^−12^ m), so the shorter clearing time is not necessarily surprising. However, as shown in Figure 3c a substantial number of 2000 nm beads remained in the siphons even after dosing at 0.01 × 10^−12^ m. At this time, fluorescence signal from the 2000 nm beads was minimal in the other organs (Figure S5e,f, Supporting Information). The mussels dosed with 200 nm beads were dissected after clearing for 21 d (Figure 2a, and Figure S4, Supporting Information). Minimal fluorescence was evident in the mussel organs (Figure S5a,b, Supporting Information), although this could be due to the lower dye loading (See note in Experimental Section).

After imaging the organs, the number of beads remaining in the mussels was quantified by fluorescence spectroscopy (Table S2, Supporting Information). In general, the mussels were able to clear a majority of the beads taken up in 24 h (Table S1, Supporting Information). In the 200 nm studies, five mussels retained an average of 5 × 10^7^ ± 2 × 10^7^ beads. This compares with 1 × 10^10^ and 2 × 10^8^ beads taken up in 24 h, respectively, showing that the mussels could clear most of the 200 nm beads. With 1000 nm beads, an average of 5 × 10^7^ ± 3 × 10^7^ beads were trapped in seven mussels. Comparatively, in 24 h the mussels took up between 2 × 10^8^ and 1 × 10^9^ 1000 nm beads. The three mussels dosed with 2000 nm beads were able to remove >99% of the beads. They retained an average of 5 × 10^5^ ± 2 × 10^5^ beads compared to 1 × 10^7^ beads taken up in the 24 h uptake study.

Despite the high clearance rates for all bead sizes (>90% in most cases, and >99% in some), at least 10^5^ to 10^7^ beads were retained in the mussels. It is possible that given enough time the mussel would be able to clear more of the beads. But, even this level of retention raises concerns about biomagnification of nanoplastics up the food chain.

### 2.3 Bioaccumulation: Internal Concentration of Beads was Less Than or Equal to the Media Concentration

We carried out this study to assess the extent to which the mussels bioaccumulate the beads—that is, increase the internal concentration of beads as compared to the bead concentration in the water. The results are summarized in Table S3 (Supporting Information). At 1 × 10^−12^ m, the mussels did ingest ≈2–10× more 1000 nm beads over 3 d as compared to a 24 h exposure (10^9^ beads vs 10^8^ beads). With 1000 nm beads at 0.1 × 10^−12^ m, the mussels ingested comparable numbers of beads (≈10^8^) whether or not extra beads were added every 24 h. Similar trends were observed in a group exposed to 2000 nm beads at 0.01 × 10^−12^ m. When new 2000 nm PS beads were added every 24 h, the mussels took up 3 × 10^7^ and 1 × 10^8^ beads over 72 h. This is only slightly higher than the 24 h exposure, which resulted in uptake of 1 × 10^7^ PS beads (Table S1, Supporting Information). Calculation of the concentration of beads in the mussels revealed that, in general, little to no bioaccumulation occurred.

### 2.4 PS Beads were Concentrated and Retained in the Siphons

In both the uptake and clearance studies, the PS beads concentrated in the siphons (Figures 1c,f,i and 3). While the mussels may have been able to actively expel the beads from other tissues, the beads became trapped in the siphons. Figure 3 particularly illustrates the accumulation of 1000 and 2000 nm beads in the siphons during the during the clearance studies even when the gills were substantially cleared (Figure S5, Supporting Information) as compared to the uptake studies (Figure 1 and Figure S1, Supporting Information). In fact, after dosing with the 1000 and 2000 nm beads, the siphons were visibly pink to the naked eye (Figure S6, Supporting Information). The mechanism by which the beads became trapped in the siphons is unknown, but it is surprising given that foreign material enters and exits through the siphons. As mentioned above, mussels can immediately reject ingested material as pseudofeces through the inhalant siphon. If some of the excreted beads become trapped in the siphon tissue instead of being fully released, this accounts for, in part, the accumulation of PS beads in the siphons. Mussels excrete foreign material that passes all the way through the digestive tract as feces through the exhalant siphon, again providing opportunities for the material to become trapped. Finally, mussels extend their siphons into the water, increasing exposure of those tissues to environmental beads (See Table of Contents figure).

The concentrations of environmental microplastics (and likely nanoplastics) are low enough such that isolating and characterizing them presents a substantial analytical challenge. As our results presented here demonstrate, retention of beads in the siphons of filter feeders is the best concentration mechanism we have found to date. In particular, the accumulation and retention of high concentrations of plastics in mussel siphons provides a unique handle by which to identify and characterize environmental exposure to aquatic plastics. However, environmental plastics are unlikely to be fluorescent like the beads used in these laboratory studies and high‐throughput, high‐confidence detection and quantification remains a paramount challenge.

### 2.5 Morphological and Chemical Identification of PS Beads by Atomic Force Microscopy‐Infrared Spectroscopy (AFM‐IR) and Photothermal Infrared (PTIR)

**Figure 4:**
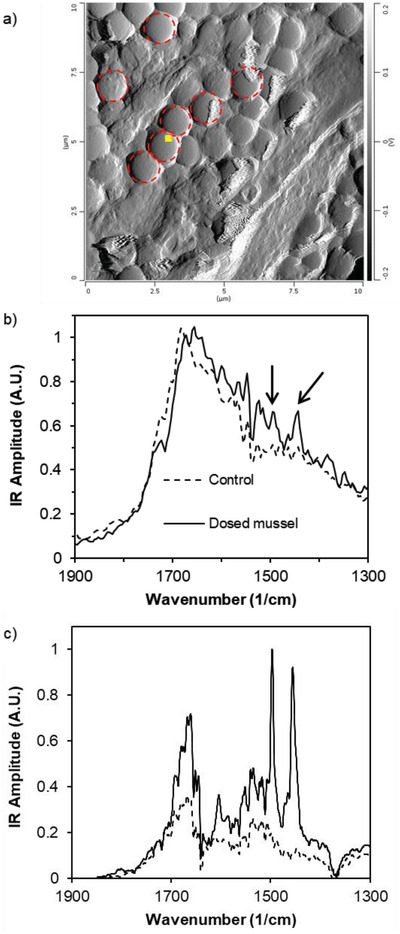
a) AFM deflection image of 1000 nm PS beads in a siphon from a quagga mussel dosed with beads at 1 × 10^−12^ m (see Figure S7, Supporting Information, for AFM images of undosed mussel siphons). The small yellow square indicates where the AFM‐IR spectrum was acquired. Dotted red circles highlight representative PS beads; b) AFM‐IR spectra comparing mussel siphon with PS beads and control siphon. Distinctive PS peaks at 1452 and 1492 cm^−1^ are evident in the spectrum of the dosed mussel (indicated with arrows); c) mIRage IR spectrum of a mussel siphon embedded with PS beads. The solid line corresponds to a region of the siphon with PS beads. The distinctive PS signals at 1452 and 1492 cm^−1^ are obvious. The dashed line corresponds to a region of the siphon with no PS beads.

The ability to detect PS beads using morphological and chemical means is necessary for in situ specimens because environmental nanoplastics are likely not fluorescent. AFM‐IR allowed for both morphological and chemical characterization of 1000 nm PS beads in the mussel siphons. Beads were easily identifiable in the AFM images (**Figure** **4**a and Figure S7a,b, Supporting Information) and in the IR spectra (Figure 4b) in the siphons from mussels dosed at 1 × 10^−12^ m. The IR spectra of the PS beads in the siphons show characteristic PS signals at 1452 and 1492 cm^−1^. Beads could not be identified in the mussel siphons exposed to lower concentrations of beads. It is possible this is not due to the absence of beads entirely but rather because the randomly chosen imaging locations did not include beads. AFM can only sample a small portion of a (macroscale) surface at a time, in the case of these images 20–30 µm. It would be prohibitively time consuming to image the entirety of the mussel siphons, so best attempts were made to achieve representative imaging of the samples. We imaged ≈6–10 locations per mussel siphon depending on how easy it was to locate the beads.

One of the characteristics of AFM‐IR is that the IR analysis includes only a small sample volume at each location, usually 10–30 nm laterally and 30–60 nm vertically. This means that in many cases the technique only provides chemical characterization data on the surface layer of a sample. If the PS beads are embedded under the top layer of siphon tissue or covered in biofilm, as is likely the case with environmental micro‐ and nanoplastics, their characteristic chemical signatures may not be observable by AFM‐IR because of the technique's limited vertical sample volume. What would likely be detected by AFM‐IR is the chemical signatures of the top layer of tissue of biofilm and not the chemical signatures of the PS beads. In general, the IR spectrum of tissue is similar to a “standard” protein spectrum with the predicted amide I, II, and III bands. This is exactly what we observed.

PTIR spectroscopy addresses the challenge of characterizing the chemical signatures of material below the biofilm and may prove a better technique for studying environmental nanoplastics. This technique achieves sub‐micrometer IR resolution and detects signal over a much larger volume. The interference of biofilms and convolution of the PS signals with the siphon tissue signals was avoided, as shown in Figure 4c. The characteristic PS signals at 1452 and 1492 cm^−1^ are very strong. These AFM‐IR and PTIR analyses illustrate the potential of these techniques to identify and characterize environmental nanoplastics in mussels.

As presented in this report, taking advantage of accumulation in sentinel filter feeders appears to be the most promising concentration method of environmental nanoplastics to date. In particular, we demonstrated that PS beads concentrate in the siphons, which provides guidance on finding and detecting nanoplastics in environmental samples. We are currently carrying out studies looking for nanoplastics in environmental mussels collected from known polluted “hot spot” sites and reference sites.

The field of research on aquatic nanoplastics is largely undeveloped because of the difficulties in isolating the polluting material. The vast majority of peer reviewed data on aquatic sub‐millimeter plastics results from plankton net trawls capturing particles in the size range of tens to hundreds of micrometers. It is likely that these studies underestimate the concentration of aquatic nanoplastics. The existing work in this area has shown that nanofibers are likely much more prevalent than particulate nanoplastics,^8^ and work examining both environmental and laboratory fibers is ongoing. Additional research studying the effect of nanoplastics morphology and surface chemistry is also needed.

## 3 Experimental Section

*Materials*: All materials were purchased from commercial sources and used as received, unless otherwise noted. FluoSpheres (fluorescent PS beads) containing a red dye (580/605 excitation/emission) and carboxylate‐modified surface were purchased from ThermoFisher Scientific. The following combinations of bead concentrations and sizes were used: 1 × 10^−12^ m for 200 nm; 1 × 10^−12^ m for 1000 nm; and 0.01 × 10^−12^ m for 2000 nm. Due to the cost of the 2000 nm PS beads, all of the experiments with this bead size were performed at 0.01 × 10^−12^ m, instead of 1 × 10^−12^ m.

*Mussel Source and Mussel Husbandry*: Quagga mussels were harvested by National Atmospheric and Oceanic Administration vessels using a Ponar grab from sites in Lake Michigan (45 m depth: 43°11.421, −86°25.724; and 90 m depth: 43°11.999, −86°31.028) and Lake Huron (45 m depth: 45°05.465, −83°04.893; 90 m depth: 45°05.541, −82°57.272). The mussels were packed in wet paper towels, transported in coolers, and then transferred to 38 L glass holding tanks. The culture media for the mussels is a simplified hard water variation of the COMBO media previously described.^44^ 2 mL of each of the following stock solutions was added per liter of distilled water: CaCl_2_ · 2H_2_O: 55.14 g L^−1^; MgSO_4_ · 7H_2_O: 55.45 g L^−1^; and NaHCO_3_: 63.0 g L^−1^. Mussels were fed RotiGrow Nanno (*Nannochloropsis*) (Reed Mariculture; Campbell, CA). An algal food solution (1 mL of RotiGrow diluted in 1 L of mussel media) was administered to the mussels in the holding tank dropwise via a feeding bag. This gradual addition prevented the food concentration from getting too high, which can cause the mussel gills to clog and therefore interfere with filtering. The holding tank mussels were fed three times per week, unless their tank still appeared cloudy and green on a feeding day–at which point that day was skipped to give the mussels time to clear the water. The mussels were kept in the holding tanks until selected for experimental trials, for a maximum of 45 d.

*Laboratory Uptake Experiments—24 h Exposure, General Procedure; See Noted Figures and Tables S1–S3 (Supporting Information) for Details on Mussel Numbers, Replicates, and Bead Concentrations*: The laboratory uptake trials were conducted using groups of three mussels housed in beakers containing 100 mL of culture media. Before adding the mussels to the beakers, the target size and concentration of fluorescent PS beads were added, as well as 2 mL of the food solution (to promote active filtering). Three mussels were selected at random from the holding tank and placed in the beakers containing the PS beads. All experiments were conducted at room temperature (22 °C). After a 24 h exposure to the PS beads, the mussels were treated in one of the following ways, depending on the experiment. (Note that when results tables contain data for only two mussels, it is because one died over the course of the experiment.)

*Dissection of Mussels and Fluorescence Microscopy*: Three beakers containing three mussels each—nine mussels total—were exposed to one of three PS bead sizes (Figure 1 and Figure S1, Supporting Information). Data are reported for eight mussels due to death of one mussel. These mussels were dissected to isolate target structures and organs for analysis. Gills, siphons, digestive tract/gonads, foot/byssal threads, and the rectum (when identifiable) were separated and placed on microscope cover slips. The organs were imaged on an Olympus IX81 fluorescence microscope. The source was a 130 W Mercury Vapor Short Arc, DC‐powered lamp, and a red color separation filter was used to characterize the fluorescence in the PS beads.

*Digestion of Mussels and Fluorescence Spectroscopy*: Three beakers containing three mussels each—nine mussels total—were exposed to one of three PS bead sizes (Table S1, Supporting Information). In order to quantify plastic beads within all tissues in aggregate, the mussels were digested following protocols modified from Dehaut et al.^45^ and Rochman et al.^17^ Briefly, the mussels were removed from the beaker and placed in the freezer for 48 h. The mussels were frozen and thawed to aid in separating the mussel tissue from the shell. The mussel tissue was placed in 40 mL 10% KOH at 60 °C and agitated gently (60 rpm). The amount of time necessary to fully digest the tissue ranged from 2 to 6 d. If large pieces of tissue were not digested, they were manually cut into smaller pieces or the tube was gently shaken. The tube was then placed back on the shaker at 60 °C for another 24 h to ensure the mussel entirely dissolved. When all the organic material had been digested, the pH of the digestate was adjusted to 6–8 using 5 m HCl. A sample from the digestate solution was analyzed by fluorescence spectroscopy. The analysis of this subsample was extrapolated to the entire digestate solution.

*Bead Clearance*: Beakers of three mussels each were exposed to one of three PS bead sizes (Figures 2 and 3, Figure S4, Table S2, Supporting Information). One treatment group had two replicate beakers (for a total of six mussels exposed to this PS bead size); one treatment group had three replicate beakers (nine mussels exposed to this PS bead size). In total, 18 mussels were treated in this experiment; data are reported for 15 mussels due to the death of 3 mussels.

To assess how long the plastic beads were retained in tissues after exposure, six groups of three mussels each were rinsed five times with culture media. Each group of three mussels was then placed in its own clean beaker containing 100 mL of fresh media and 2 mL of the algal food solution. The mussel feces and pseudofeces were collected using a P1000 micropipette every 24 h for the first 7 d, and then every other day thereafter. The rate of collection was decreased because after the first 7 d the changes in fluorescence over 24 h were minimal. After each collection event, the mussels were given fresh media and given 2 mL of food on their regular schedule of 3× week^−1^. A randomly selected subset of the collected feces was imaged by fluorescence microscopy to qualitatively analyze the rate at which the mussels were excreting the PS beads. This process was continued until at least three consecutive imaging measurements showed only baseline fluorescence comparable to the fluorescence in feces from control mussels. Some of the images are overexposed because the same microscope settings were used to collect all images. This was done to allow for quantification of the fluorescence between images. ImageJ (NIH) was used to quantify the mean fluorescence intensity in each of the feces images and boxplots produced to show the trends. Regions of interest of 100 × 100 pixels were used. The results were compiled into box plots, demonstrating the trend of decreasing fluorescence in the feces as the mussels cleared the beads.

When the feces were no longer fluorescent, the mussels were dissected and imaged via fluorescence microscopy to look for remaining fluorescent material retained within the mussels. The siphons, gills, and rectums (if they could be successfully dissected separately) were examined for remaining PS beads. After the imaging was completed, the slides were scraped, and the mussels digested as described above for quantification by fluorescence spectroscopy.

*Laboratory Uptake Experiments—72 h Exposure*: (Four treatment groups of three mussels each. Data reported for 11 mussels.) These laboratory uptake experiments were carried out under the same initial conditions as described in the 24 h experiments, but the total exposure time was extended to 72 h. The mussels were dosed at concentrations of 1.0 and 0.1 × 10^−12^ m for the 1000 nm beads and 0.01 × 10^−12^ m for the 2000 nm beads. To ensure that the mussels were not clearing all the beads from the beakers and to keep the bead concentration high, experimental groups were included in which the mussels were dosed with the original number of beads at the 0, 24, and 48 h time points. Thus, these groups were offered 3× the original number of beads as were the 24 h exposure groups.

*Fluorescence Spectroscopy of Digested Tissue*: All fluorescence spectroscopy analyses were performed on a Varian Cary Eclipse Fluorescence Spectrophotometer. The excitation wavelength of the red dye in the PS beads was 580 nm and the emission was recorded from 600 to 650 nm. The spectrum was recorded in triplicate and averaged. If the mussel digestate was cloudy or too concentrated for fluorescence spectroscopy, it was diluted with nanopure water until it was clear and colorless before the measurements were carried out. In these cases, accounted for the additional volume when calculating the concentration of PS beads in the mussels.

Calibration curves of fluorescence intensity as a function of concentration were used to calculate the concentration of PS beads in the mussels. To control for potential degradation of the PS beads or the fluorescent dye during the experiment and base digestion process, a control solution of PS beads was concurrently exposed to exactly the same conditions as the mussels . New calibration curves were created for every experiment because of the variations in experimental conditions.

Mussels collected immediately following the 24 h exposure were used to quantify average uptake of PS beads. Mussels collected at multiple time points following the exposure period were used to determine the extent to which the mussels were clearing the PS beads. In both cases, fluorescence intensity of mussel digestate was measured and the number of beads detected were quantified against a calibration curve.

*AFM‐IR of Dissected Mussel Siphons*: AFM‐IR is a technique that combines the topographical analysis of AFM with IR spectroscopy. The experiments were carried out on a nanoIR2 (Anasys Instruments, Santa Barbara, CA). The IR spectrum was generated by analyzing the oscillations of the AFM cantilever, which are dependent on the local thermal expansion of the sample. The resolution of the IR spectra is sample dependent (based on thermal transport properties), but generally is ≈30–50 nm.

Groups of three mussels were dosed at 1 × 10^−12^, 1 × 10^−15^, and 1 × 10^−18^ m with 1000 nm beads for 24 h and then allowed to clear in clean beakers for 14 d. The mussel was allowed to clear for 14 d based on the results from the bead clearance studies. At 14 d, the fluorescence level in the feces had dropped below the point of oversaturation, but there were beads remaining in the mussels. The mussels were dissected and the siphons isolated, including the inhalant, exhalant, and the tissue connecting the two. The siphons and connective tissue were visibly pink indicating the presence of beads. The siphons were allowed to dry on glass cover slips at 4 °C and then imaged by AFM‐IR. AFM imaging was carried out on nanoIR2 Contact Mode nIR2 probes (gold‐coated silicon cantilever, nominal radius 25 nm, force constant 0.07–0.4 N m^−1^, resonance frequency 13 ± 4 kHz). Line scan rates were 1 Hz, and the resolution was 512 pixels/line. Experimental spectra are an average of 32 scans. Savitzky‐Golay smoothing (polynomial order = 7, side points = 5) was applied to the raw spectra scans, which were then normalized with the maximum at 1676 cm^−1^ and averaged.

*PTIR Spectroscopy of Dissected Mussel Siphons*: PTIR spectroscopy was carried out on a mIRage IR microscope (Anasys Instruments). This technique achieves sub‐micrometer IR resolution and detects signal over a much larger volume. The same samples analyzed by AFM‐IR were used in the PTIR analyses.

*Notes on Fluorescent Polystyrene Beads*: The PS beads of different sizes have different dye loadings. That is, the bigger beads contain more dye molecules. A given number of beads with a larger diameter will appear brighter than the same number of beads of a smaller diameter. Therefore, the fluorescence intensity in the images cannot be taken as an indicator of the number of beads present; the fluorescence microscopy images provide qualitative information on whether beads are present and where they tend to concentrate in the mussel. This effect is likely observed comparing Figure 1a,d,g. Very few 200 nm beads appear to be present in the mussel gills (Figure 1a), as compared with the other two bead sizes. This could be due in part to a lack of retention of 200 nm beads on the gills but could also be attributed to a lower dye loading of the beads. For a quantitative analysis, fluorescence spectroscopy of digested samples was used to determine the number of beads taken up and retained in the mussels.

As discussed, substantial uptake of the 2000 nm beads was still evident at these lower concentrations because (1) there were more dye molecules per bead, and (2) 2000 nm is in the preferred food size range.

## Conflict of Interest

The authors declare no conflict of interest.

## Supporting information

Supporting Information
