# Supplementary material for: Uptake and Retention of Nanoplastics in Quagga Mussels
Source: Glob Chall. 2019 Jul 2;4(6):1800104. doi: 10.1002/gch2.201800104 (PMC7268195; doi:10.1002/gch2.201800104)
Supplement: Supplementary file 1 — Supporting Information [file GCH2-4-1800104-s001.pdf]

Copyright WILEY-VCH Verlag GmbH & Co. KGaA, 69469 Weinheim, Germany, 2020.

Global Challenges

Supporting Information

for *Global Challenges,* DOI: 10.1002/gch2.201800104

Uptake and Retention of Nanoplastics in Quagga Mussels

Rachel L. Merzel, Lauren Purser, Taylor L. Soucy, Monica Olszewski, Isabel Colón-Bernal, Melissa Duhaime, Ashley K. Elgin,* and Mark M. Banaszak Holl**

Supporting Information for

Uptake and retention of nanoplastics in quagga mussels

Rachel L. Merzel,[1a] Lauren Purser,[1a] Taylor L. Soucy,[1a] Monica Olszewski,[1] Isabel Colón-Bernal,[1] Melissa Duhaime,[2*] Ashley K. Elgin,[3*] Mark M. Banaszak Holl[4*]

[1]Chemistry Department, University of Michigan, Ann Arbor, MI 48109, United States
[2]Ecology and Evolutionary Biology, University of Michigan, Ann Arbor, MI 48109, United States
[3]NOAA Great Lakes Environmental Research Laboratory, Lake Michigan Field Station, Muskegon, MI 49441, United States
[4]Chemical Engineering, Monash University, Clayton VIC 3800, Australia
*corresponding authors: duhaimem@umich.edu, ashley.elgin@noaa.gov, mark.banaszakholl@monash.edu

[a]Contributed equally to manuscript

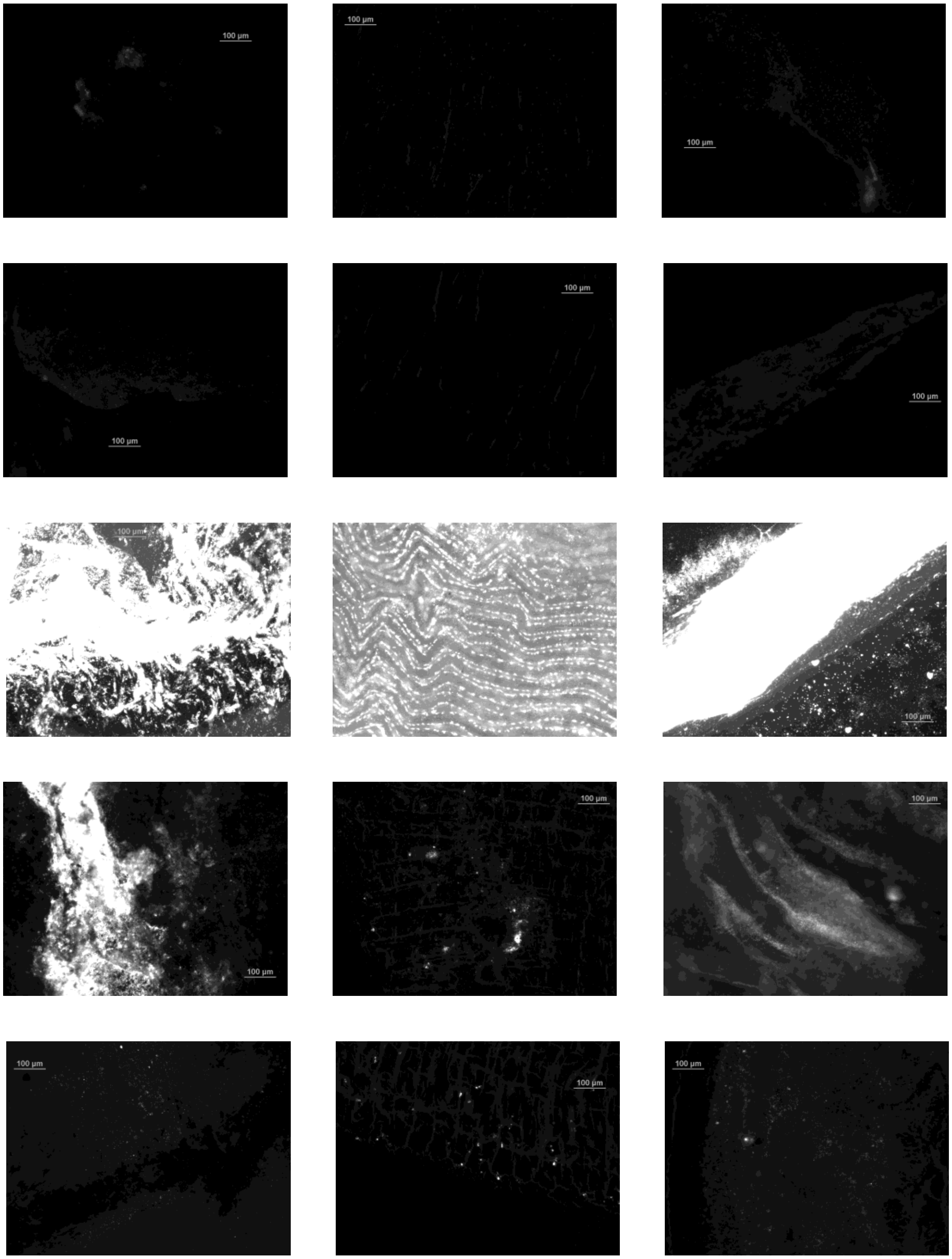

Figure S1. Fluorescence microscopy images of dissected mussel organs following 24 hour uptake studies with carboxylic acid-terminated PS beads containing a red dye.

Recently reported marine microplastic concentrations:

127,000-2,000,000 particles/km^2 in the Great Lakes

15,000-2,000,000 particles/km^2 (ave. = 700,000 particles/km^2 in the San Francisco Bay)

Using 1,000,000 particles/km^2 as a general estimate, there are 0.01 particles/L of water

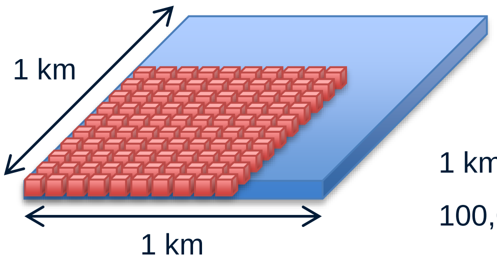

Consider the volume to arise from the number of liter volumes packed into a 1x1 km surface (effective sampling depth of 10 cm)

1 km = 10,000 1L cubes per side

100,000,000 1L cubes in 1 km^2

**Count description**	**Particles/L**	**L/particle**
**Average**	**0.01**	**100**
Low	0.00015	6,667
High	0.02	50

Figure S2. Calculations demonstrating how microplastic concentrations expressed in particles/km^2 were converted to particles/L. The table shows the calculations for how many liters of water would need to be collected to find one microplastic, based on the reported environmental concentrations.

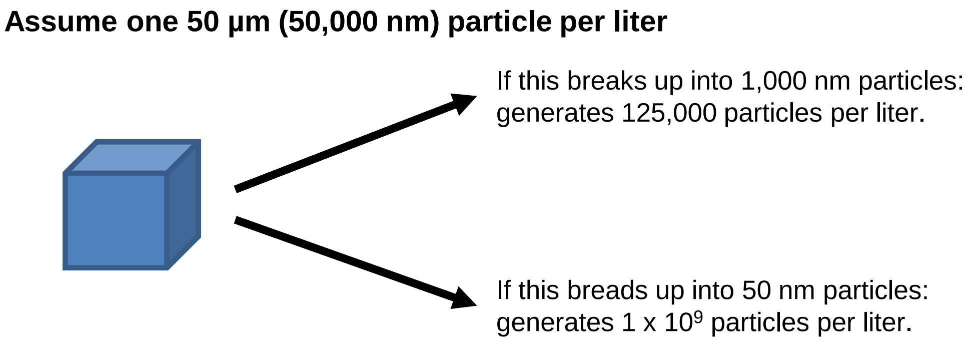

Figure S3. Schematic estimating local concentrations of nanoplastics that could be produced from the degradation and fragmentation of microplastics.

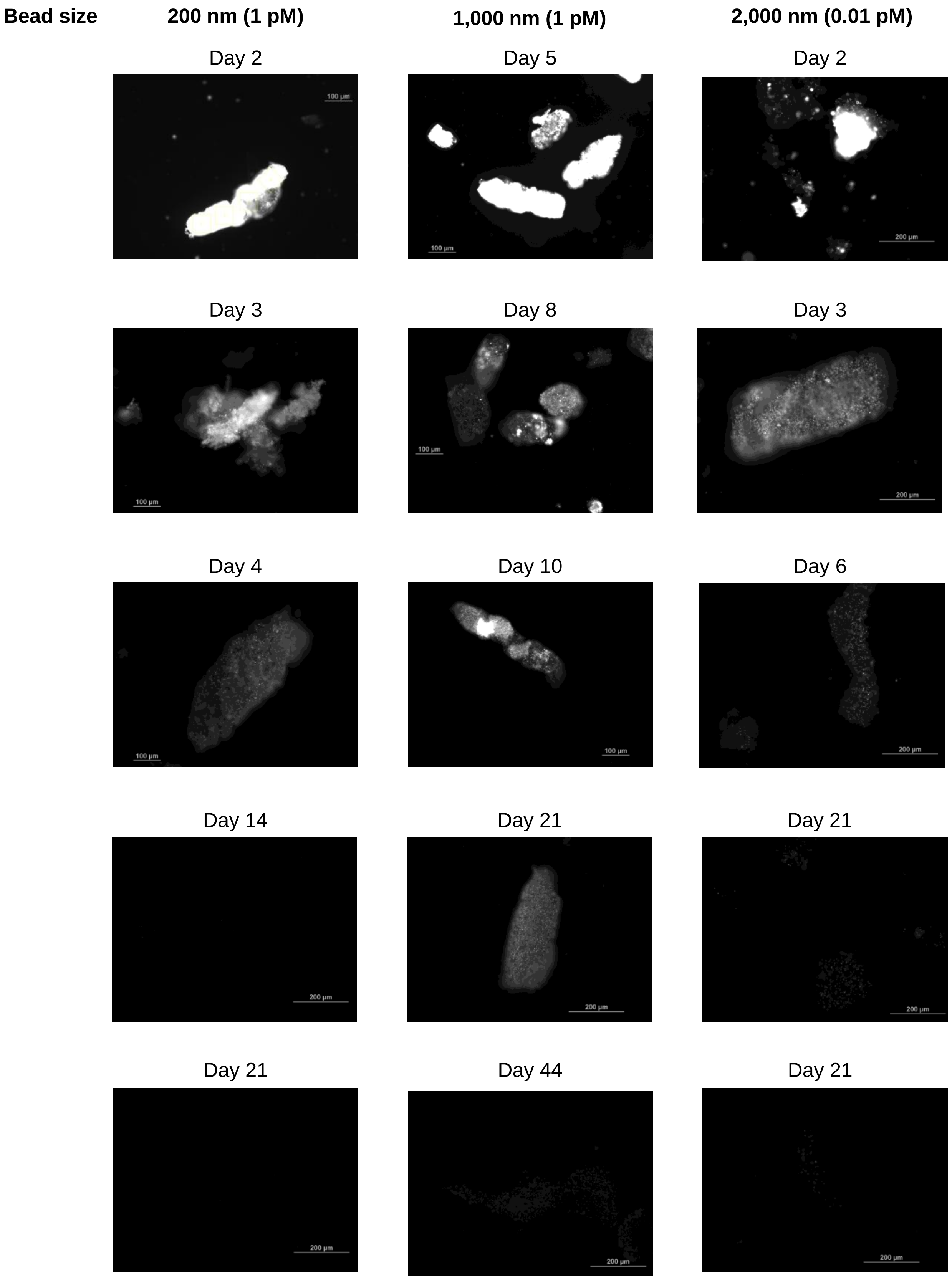

Figure S4. Fluorescence microscopy images of mussel feces following dosing with PS beads containing a red dye. The mussels were dosed for 24 hours and then given time to clear the ingested beads.

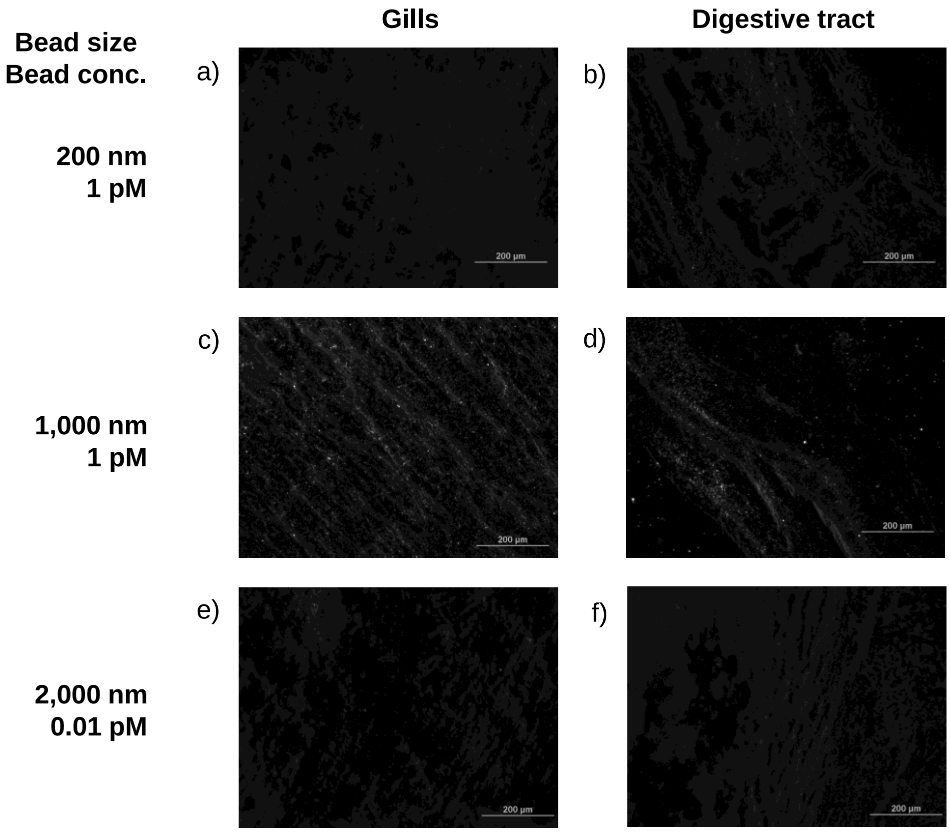

Figure S5. Fluorescence images of quagga mussel gills and digestive tract after mussels were dosed with carboxylic acid-terminated PS beads containing a red dye and allowed to clear until their feces were no longer fluorescent.

a)

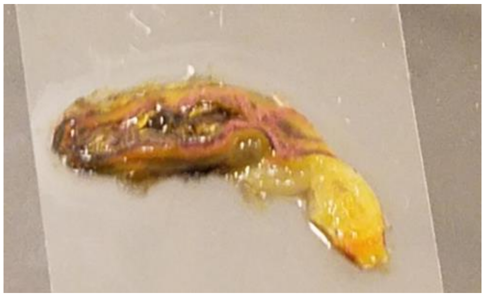

b)

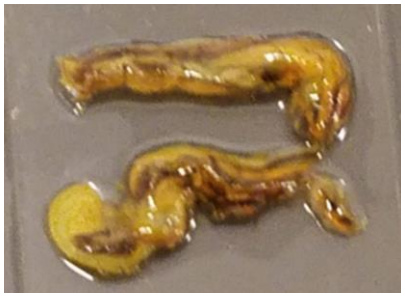

Figure S6. a) Dissected siphon from a mussel dosed with 1,000 nm beads containing a red dye. Following 24 hours of exposure, the mussel was allowed to clear the beads for 14 days before being dissected; b) Dissected siphon of a control (non-dosed) mussel.

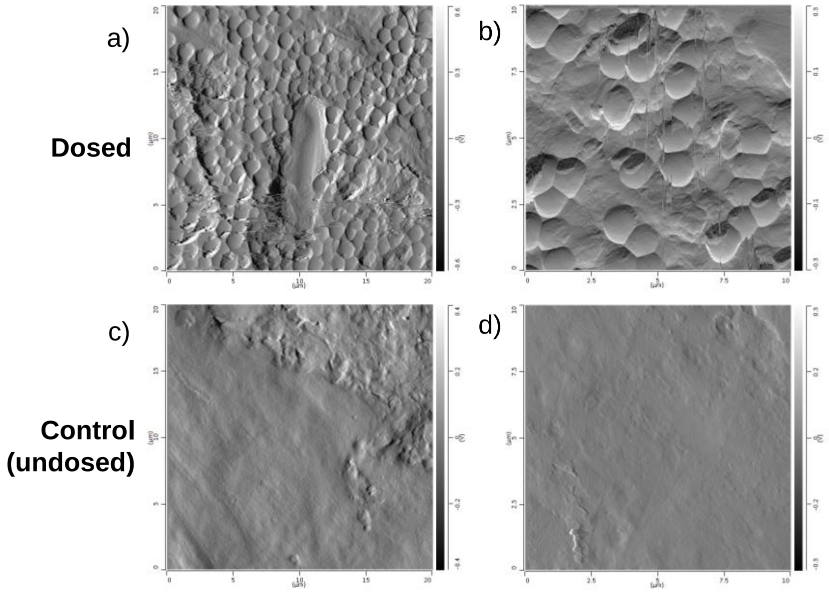

Figure S7. a) 20 x 20 µm and b) 10 x 10 µm AFM deflection images of 1,000 nm beads in a mussel siphon; c) 20 x 20 µm and d) 10 x 10 µm deflection images of control (non-dosed) mussel siphons.

Table S1. Summary of results from fluorescence spectroscopy studies quantifying the number of carboxylate-terminated PS beads mussels ingested in 24 hours. Bead concentrations were 1 pM for 200 and 1,000 nm beads and 0.01 pM for 2,000 nm beads.

Bead diameter (nm) (conc.)	Mussel	Number of beads taken up in 24 hours
200 (1 pM)	A-1	$1x10^{10}$
	A-2	$2x10^{8}$
1,000 (1 pM)	B-1	$1x10^{9}$
	B-2	$5x10^{8}$
	B-3	$2x10^{8}$
2,000 (0.01 pM)	C-1	$1x10^{7}$
	C-2	$1x10^{7}$
	C-3	$1x10^{7}$

Table S2. Summary of fluorescence spectroscopy studies quantifying the number of PS beads retained in the mussels after being given time to clear the beads.

Bead diameter (nm)	Mussel	Days allowed to clear	Ave. number beads remaining in mussels
200	D-1	21	$1x10^8$
	D-2		$6x10^7$
	E-1	17	$5x10^7$
	E-2		$1x10^7$
	E-3		$2x10^7$
1,000	F-1	44	$3x10^7$
	F-2		$3x10^7$
	G-1[b]	49	$6x10^7$
	G-2[b]		$4x10^7$
	G-3[b]	Died on day 44-46	$2x10^4$
	H-1[b]	49	$1x10^8$
	H-2[b]		$7x10^7$
	H-3[b]		$1x10^8$
2,000 (0.01 pM)	I-1	20	$4x10^5$
	I-2		$6x10^5$
	I-3		$1x10^5$

[a]Mussel was not dissected before digesting and quantifying the number of beads
[b]Replicates B&C were carried out at the same time but as separate trials in different beakers

Table S3. Summary of fluorescence spectroscopy studies quantifying the number of PS beads taken up into the mussels after 3 days of exposure to an excess of beads

Bead diameter (nm)	Nominal bead conc. (pM)	Total number beads added	Mussel	# beads in mussel	Conc. of beads in mussel (pM)
1,000	1	$1.2x10^{11}$	J-1	$2x10^{9}$	1.3
			J-2	$1x10^{9}$	0.3
			J-3	$2x10^{9}$	1.2
	0.1	$1.2x10^{10}$	K-1	$2x10^{8}$	0.1
			K-2	$1x10^{8}$	0.3
			K-3	$7x10^{8}$	0.7
	0.3 (0.1 pM added at 0, 24, 48 hrs)	$3.6x10^{10}$	L-1	$4x10^{8}$	0.2
			L-2	$3x10^{9}$	2.2
			L-3	$4x10^{8}$	0.3
2,000	0.03 (0.01 pM Added at 0. 24, 48 hrs)	$3.6x10^{9}$	M-1	$1x10^{8}$	0.08
			M-2	$3x10^{7}$	0.02
